# Biological properties of *Staphylococcus* virus ΦSA012 for phage therapy

**DOI:** 10.1038/s41598-022-25352-6

**Published:** 2022-12-09

**Authors:** Jumpei Fujiki, Tomohiro Nakamura, Keisuke Nakamura, Keita Nishida, Yurika Amano, Yusaku Watanabe, Satoshi Gondaira, Masaru Usui, Masaru Shimizu, Kazuhiko Miyanaga, Shinya Watanabe, Tomohito Iwasaki, Kotaro Kiga, Tomoko Hanawa, Hidetoshi Higuchi, Teiji Sawa, Yasunori Tanji, Yutaka Tamura, Longzhu Cui, Hidetomo Iwano

**Affiliations:** 1 Laboratory of Veterinary Biochemistry, Rakuno Gakuen University School of Veterinary Medicine, Ebetsu, Hokkaido Japan; 2 Phage Therapy Institute, Waseda University, Tokyo, Japan; 3 Research Center for Drug and Vaccine Development, National Institute of Infectious Diseases, Tokyo, Japan; 4 Laboratory of Animal Health, Rakuno Gakuen University School of Veterinary Medicine, Ebetsu, Hokkaido Japan; 5 Laboratory of Food Microbiology and Food Safety, Rakuno Gakuen University School of Veterinary Medicine, Ebetsu, Hokkaido Japan; 6 Department of Anesthesiology, Kyoto Prefectural University of Medicine, Kyoto, Japan; 7 Division of Bacteriology, Department of Infection and Immunology, School of Medicine, Jichi Medical University, Shimotsuke, Tochigi Japan; 8 Department of Food Science and Human Wellness, College of Agriculture, Food and Environment Science, Rakuno Gakuen University, Ebetsu, Japan; 9 Department of Infectious Diseases, Kyorin University School of Medicine, Mitaka, Tokyo Japan

**Keywords:** Microbiology, Antimicrobials, Applied microbiology, Bacteriophages, Phage biology

## Abstract

*Staphylococcus* virus ΦSA012 has a wide host range and efficient lytic activity. Here, we assessed the biological stability of ΦSA012 against temperature, freeze-thawing, and pH to clinically apply the phage. In addition, inoculation of ΦSA012 through i.p. and i.v. injections into mice revealed that phages were reached the limit of detection in serum and accumulated notably spleens without inflammation at 48 h post-inoculation. Furthermore, inoculation of ΦSA012 through s.c. injections in mice significantly induced IgG, which possesses neutralizing activity against ΦSA012 and other *Staphylococcus* viruses, ΦSA039 and ΦMR003, but not *Pseudomonas* viruses ΦS12-3 and ΦR18 or *Escherichia* viruses T1, T4, and T7 in vitro. Immunoelectron microscopic analysis showed that purified anti-phage IgG recognizes the long-tail fiber of *staphylococcus* viruses. Although *S. aureus* inoculation resulted in a 25% survival rate in a mouse i.p. model, ΦSA012 inoculation (i.p.) improved the survival rate to 75%; however, the survival rate of ΦSA012-immunized mice decreased to less than non-immunized mice with phage i.v. injection at a MOI of 100. These results indicated that ΦSA012 possesses promise for use against staphylococcal infections but we should carefully address the appropriate dose and periods of phage administration. Our findings facilitate understandings of *staphylococcus* viruses for phage therapy.

## Introduction

*Staphylococcus aureus*, which is a gram-positive bacterium that inhabits animals and humans, has high pathogenicity and causes severe infectious diseases. Mortality rates in humans are higher than 30% in cases of severe systemic infections^1,2^. Healthcare-associated *S. aureus* causes a variety of infections such as respiratory, urinary tract, surgical-associated, and blood stream infections through long-term hospital stays, whereas community-associated *S. aureus* causes skin and soft tissue infections in healthy people who have no association with hospital care^3^. In addition, livestock-associated *S. aureus* is one of the most frequent pathogens involved in subclinical and clinical bovine mastitis, leading to economic losses of $100 million every year due to decreased milk production in the United States and Japan. Therefore, successful prevention and treatment of *S. aureus* infections would have great impacts not only on human health, but also industry^4,5^.

Infectious diseases caused by antimicrobial-resistant bacteria have considerable negative impacts on public health. O’Neill et al*.* estimated that 10 million people will die annually due to antimicrobial-resistant bacterial infections by 2050 without a global program against antimicrobial resistance (AMR)^6^. Notably, *S. aureus* is classified as an ESKAPE pathogen (other examples of which include *Enterococcus faecium, S. aureus, Klebsiella pneumoniae, Acinetobacter baumanii, Pseudomonas aeruginosa*, and *Enterobacter spp.*) that are frequently isolated from clinical human patients and considered the major multidrug resistant bacteria^7^. Although antibiotics such as β-lactams have been used to control *Staphylococcus* infections as a standard treatment, the rapid acquisition of AMR known as methicillin-resistant *S. aureus* (MRSA) has made antimicrobial treatment of infections a significant challenge^8^. Therefore, the World Health Organization has listed *S. aureus* as a priority pathogen for the research and development of new antibiotics. In this context, phage therapy has received significant attention as an alternative approach for treating AMR infections, including those caused by *S. aureus*.

Phage therapy works by using bacteriophages, simply called phages, which are viruses that specifically infect bacteria. It has been applied to bacterial infectious diseases for a long time, notably in eastern European countries such as Russia, Georgia, and Poland^9,10^. Recent studies have shown successful application of phage therapy against AMR infections in the United States, United Kingdom, France and Belgium^11–14^. However, in general, phages have very narrow host ranges against target bacteria at the strain level, which limits their use in therapy. Despite this, it has been suggested that *Staphylococcus* virus K-like phages exhibit a wider host range at the species level^15–17^, indicating that this kind of phage could be effective against a variety of staphylococcal infections. Among *Staphylococcus* phages, ΦSA012, which was isolated by using bovine mastitis-derived *S. aureus* (SA003), has been identified as potentially useful for phage therapy^18^. It has been suggested that ΦSA012 recognizes the backbone of wall teichoic acid (WTA) as a receptor^19–21^, leading to a broad host range, because WTA is a highly conserved structure in staphylococcal species. A previous study showed that ΦSA012 has bactericidal activity against all tested *S. aureus* strains derived from bovine mastitis (93 strains from 40 genotypes) and reduces SA003 proliferation and inflammation in the mammary glands of a mouse mastitis model^18,22^. Thus, the detailed biological properties of ΦSA012 should be further studied to implement phage therapy against a wide range of staphylococcal infections.

In the present study, we reevaluated ΦSA012 classification using whole-genome sequencing and the host range by the efficacy of plating (EoP) method. We also assessed the biological stability of ΦSA012 against temperature, freeze-thawing, and pH, in addition to its pharmacokinetics and the immune response of ΦSA012 in mice. Our results indicated that ΦSA012 has promise for phage therapy and is biological stable without causing inflammation, though a high dose of ΦSA012 induces anti-phage antibodies with neutralizing activity. These results provide insight into the potential utility of *Staphylococcus* viruses for phage therapy to combat staphylococcal infections.

## Results

### Reevaluation of ΦSA012 classification and host range

**Figure 1:**
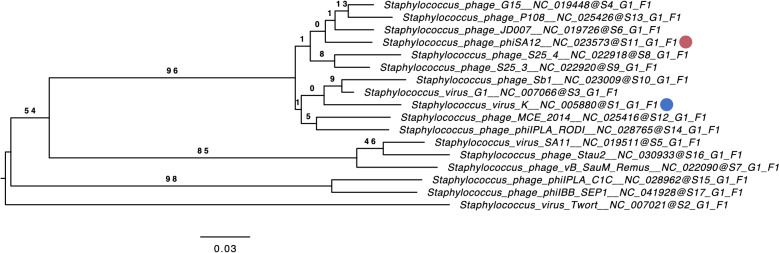
Phylogenetic analysis using the whole-genome sequence of ΦSA012. The phylogenetic tree was constructed by VICTOR using 16 whole-genome sequences of all *Staphylococcus* K-virus master species registered by the International Committee on the Taxonomy of Viruses (ICTV). The red circle indicates ΦSA012 and the blue circle indicates *Staphylococcus* virus K. S, G, and F after the phage names refer to species, genus, and family clusters, respectively. Bootstrap values are shown on the branch nodes, and the scale bar represents a distance of 0.03 substitutions per site.

Phages were previously classified by morphology, and ΦSA012 was recently categorized into the *Myoviridae* family based on microscopic analysis^18^. We thus first conducted phylogenetic analysis to clarify the genetic relationships between known *Staphylococcus* viruses and ΦSA012. A phylogenetic tree constructed using the ΦSA012 whole genome by VICTOR resulted in seventeen species, one genus, and one family cluster (Fig. 1), indicating that ΦSA012 is a *Staphylococcus* virus K-like phage. In addition, ΦSA012 harbored no undesirable genes such as those associated with bacterial virulence or drug resistance, and nor did it have temperate phage-associated genes in its whole genome (data not shown).

**Figure 2:**
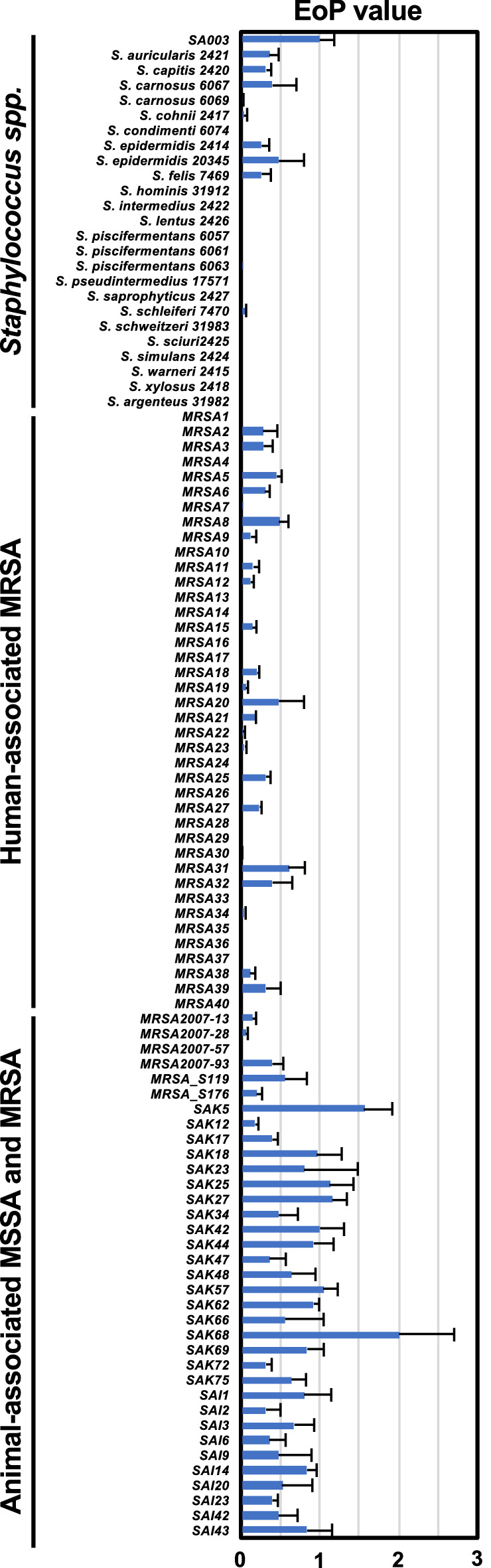
EoP of ΦSA012 against *Staphylococcus spp.* strains and *S. aureus* clinical isolates. SA003 was assigned as the control host against ΦSA012. EoP values are indicated as fold changes in the effect on SA003 and are presented as means ± SD (n = 3).

To address the ΦSA012 host spectrum, we performed EoP assays, because the previously reported ΦSA012 host spectrum was only assessed by single spot test^18,22^, which had the possibility of overestimation by lysis from without^23^. As shown in Fig. 2, ΦSA012 possesses plaque formation activity against 94.4% (33/35) of animal-associated MRSA and MSSA and against 60.0% (24/40) of human-associated MRSA. Although ΦSA012 revealed higher EoP values against almost all animal-derived MSSA compared to SA003, ΦSA012 resulted in lower EoP values against almost all MRSA compared to that against SA003. In addition, ΦSA012 was active against 41.7% (10/24) of *Staphylococcus spp.* such as *S. auricularis*, *S. capitis*, *S. carnosus*, *S. cohnii*, *S. epidermidis*, *S. felis*, *S. piscifermentans*, and *S. schleiferi*. EoP values against these *Staphylococcus spp.* were lower than that against SA003.

### ΦSA012 stability against various environmental conditions

**Figure 3:**
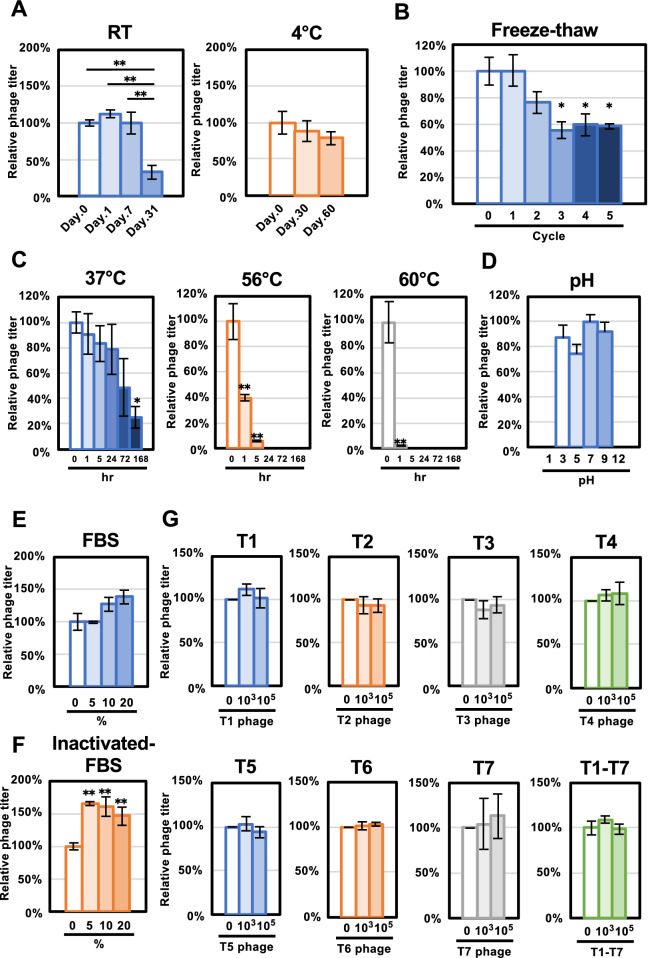
ΦSA012 stability against several environmental conditions. (**A**) Phage stability against storage conditions at room temperature and 4 °C. ΦSA012 infectivity is indicated as fold changes compared to day 0 and is presented as means ± SD (n = 3). (**B**) Environmental resistance of ΦSA012 against freeze–thaw cycles. The titer of ΦSA012 is indicated as the fold change compared to cycle 0 and is presented as means ± SD (n = 3). (**C**) Phage stability at temperatures of 37 °C, 56 °C, and 60 °C. The titer of ΦSA012 is indicated as the fold change compared to 0 h and is presented as means ± SD (n = 3). (**D**) Phage stability against different pH ranges. The titer of ΦSA012 is indicated as the fold change compared to pH 7 and is presented as means ± SD (n = 3). (**E** and **F**) Effects of serum on the plaque-forming activity of ΦSA012. The titer of ΦSA012 is indicated as the fold change compared to 0% FBS and is presented as means ± SD (n = 3). (**G**) Effects of other phage particles on the plaque-forming activity of ΦSA012. The titer of ΦSA012 is indicated as the fold change compared to non-treated ΦSA012 and is presented as means ± SD (n = 3). The significance compared to the control under each condition was analyzed by Tukey’s test based on one-way ANOVA: *p < 0.05, **p < 0.01.

We initially assessed ΦSA012 stability under several storage conditions. As shown in Fig. 3A, the ΦSA012 titer was stable after 2 or 7 days of incubation at room temperature, but decreased significantly and was under 50% at 31 days. In contrast, the 4 °C storage condition did not affect the viral titer of ΦSA012 significantly at 30 and 60 days; however, monitoring of the OD value clearly showed that attenuated phage aliquots that were stored at room temperature for 31 days resulted in efficient lytic activity at 7 hpi, comparable with day 0 phages (Fig. S1A). In addition, we measured the effects of freeze–thaw cycles on ΦSA012 titers as shown in Fig. 3B. The 1st cycle had no effect on the plaque-forming activity of ΦSA012, but the ΦSA012 titer decreased to less than 80% after the 2nd cycle. In the 3rd, 4th, and 5th cycles, the ΦSA012 titer decreased significantly compared with the non-freeze–thaw control phages, to around 60%.

Next, we assessed ΦSA012 stability following exposure to varying temperatures and pH. As shown in Fig. 3C, the ΦSA012 titer decreased gradually in a time-dependent manner at 37 °C. After 168 h of incubation at 37 °C, the ΦSA012 titer was reduced to around 20% compared to 0 h; however, while ΦSA012 attenuated to 20% of the viral titer and took a long time to inhibit bacterial growth compared to non-treated ΦSA012, it still had efficient lytic activity against SA003 after 6 hpi, which was the same as the non-treated ΦSA012 lytic curve (Fig. S1B). In contrast, incubations of 24 h and 5 h were lethal to ΦSA012 at 56 °C and 60 °C, respectively, according to plaque assays (Fig. 3C) and lytic curves (Fig. S1B). To evaluate ΦSA012 resistance to pH, we exposed it to specific pHs for 1 h. Although ΦSA012 had diminished plaque-forming activity in pH 1 and pH 11 buffers, it was still active against SA003 at pH ranging from 3 to 9. Compared to pH 7, the ΦSA012 titer decreased 10–20% at pH 3, 5, and 9 (Fig. 3D), but this was not significant. We detected similar lytic activities of ΦSA012 that were treated at pH 3, 5, 7 and 9 when monitoring the OD value (Fig. S1C).

Furthermore, we determined ΦSA012 resistance to biological factors such as serum and other phage particles. FBS with or without inactivation did not attenuate the ΦSA012 titer in 5%, 10%, and 20% conditions (Fig. 3E and F). To assess the effect of other phage particles on ΦSA012, we mixed T1-T7 phages (10^3^ or 10^5^ pfu/mL) with ΦSA012 (10^5^ pfu/mL). As shown in Fig. 3G, T1-T7 phages did not inhibit ΦSA012 plaque-forming activity.

### Pharmacokinetics of ΦSA012 in mice

**Figure 4:**
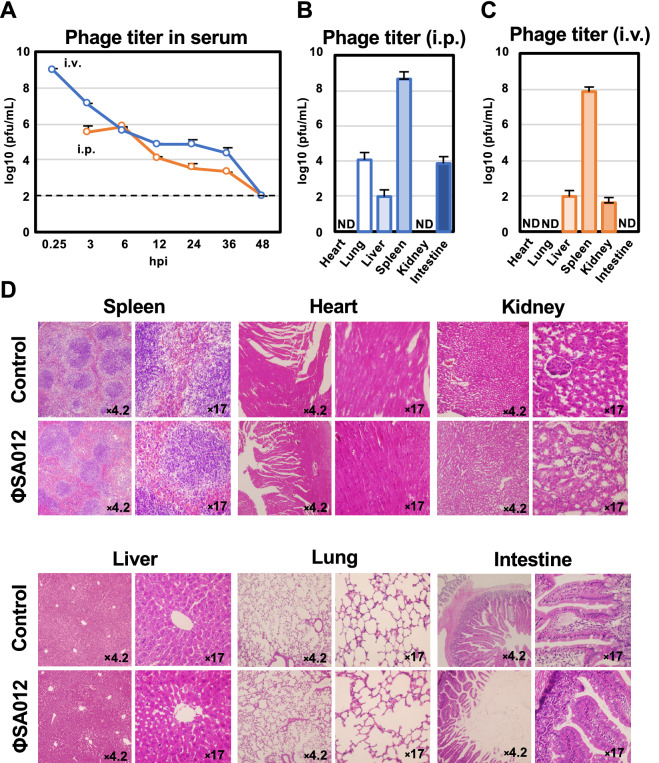
Pharmacokinetics and side effects of ΦSA012 in mice. (**A**) Pharmacokinetics of ΦSA012 administered with i.p. and i.v. injections into mice. Phage titers in the serum were monitored from 3 to 48 hpi for i.p. and from 0.25 hpi to 48 hpi for i.v. injections. The titers of ΦSA012 in serum samples are presented as means ± SD (n = 3). 10^2^ pfu/mL is the limit of detection of the phage in the plaque assay. (**B**) Accumulation of ΦSA012 in organs at 48 hpi after i.p. injection. The titers of ΦSA012 in organ homogenates are presented as means ± SD (n = 3). (**C**) Accumulation of ΦSA012 in organs at 48 hpi after i.v. injection. The titers of ΦSA012 in organs homogenates are presented as means ± SD (n = 3). ND means that plaques were not detected, indicating values less than 1.2 × 10^2^ pfu/g in the heart, 8.9 × 10^1^ pfu/g in the lung, 4.5 × 10^2^ pfu/g in the kidney, and 8.7 × 10^1^ pfu/g in the intestine. (**D**) Histopathology and pathological changes of organs in ΦSA012-inoculated mice (i.p.). The spleens, hearts, kidneys, livers, lungs, and intestines were stained with hematoxylin and eosin. The number in each picture represents magnification.

The pharmacokinetics of ΦSA012 in mouse serum were evaluated after i.v. or i.p. phage inoculation. As shown in Fig. 4A, phages in serum decreased in a time-dependent manner following i.v. or i.p injections. Notably, the phage titers reached their highest level after 15 min and 6 h after i.v. or i.p injections, respectively, and reached their limit of detection after 48 h. At 48 hpi, ΦSA012 had mostly accumulated in the spleens after i.v. or i.p injections (Fig. 4B and C). Phages were also detected in the liver, lung, and intestine, but not in the heart (Fig. 4B and C). In addition, we investigated the potential side effects of ΦSA012 on organs. As shown in Fig. 4D, tissue samples from ΦSA012-inoculated mice at 48 hpi did not reveal any inflammation or pathological changes.

### Immune response against ΦSA012 in mice

**Figure 5:**
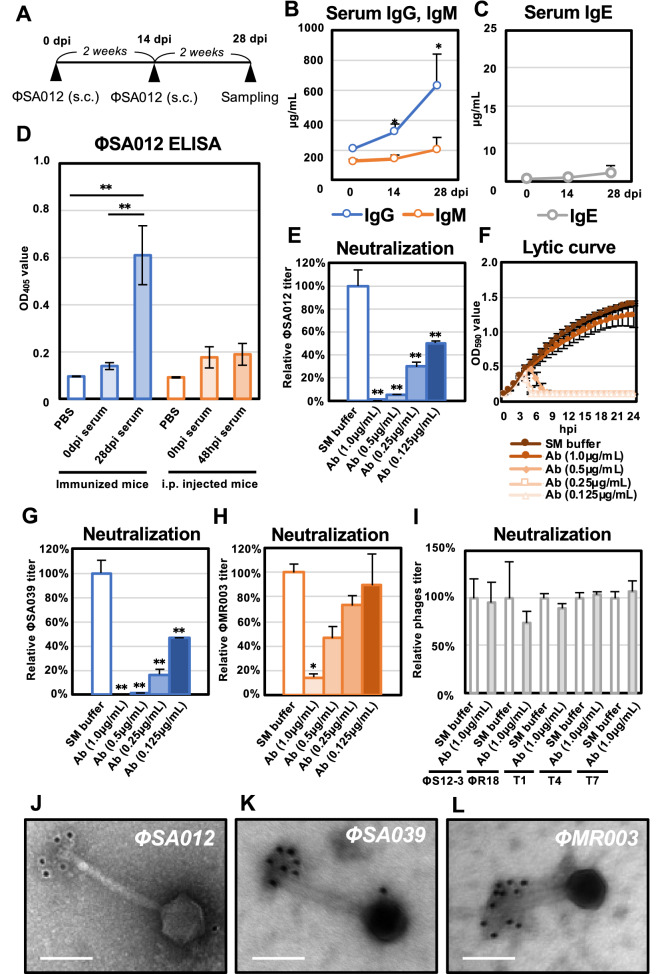
Immune response in mice to ΦSA012. (**A**) Time course of ΦSA012 immunization in mice by s.c. injection. (**B**) Concentrations of IgG and IgM in serum samples from immunized mice measured using ELISA. Data are presented as means ± SD (n = 4). Significance against 0 dpi was analyzed by Tukey’s test based on one-way ANOVA: *p < 0.05, **p < 0.01. (**C**) Concentrations of IgE in serum samples from immunized mice measured using ELISA. Data are presented as means ± SD (n = 4). (**D**) Anti-phage antibody responses against ΦSA012. Serum samples from mice immunized with s.c. injections or inoculated with i.p. injections of ΦSA012 were tested for phage-specific IgG responses by ELISA. Data are presented as means ± SD (n = 4). PBS was added as a control instead of serum samples in the phage ELISA. Significance among each group was analyzed by Tukey’s test based on one-way ANOVA: *p < 0.05, **p < 0.01. (**E**) Neutralization activity of purified anti-phage IgG against ΦSA012. The titers of ΦSA012 are indicated as fold changes against the control and presented as means ± SD (n = 3). Significance against control was analyzed by Tukey’s test based on one-way ANOVA: *p < 0.05, **p < 0.01. (**F**) Lytic curves of SA003 growing in the presence of ΦSA012 treated with purified anti-phage IgG were obtained by monitoring the OD_590_ until 24 hpi. The individual points in each lytic curve are presented as means ± SD (n = 4). (**G** and **H**) Neutralization activity of purified anti-phage IgG against ΦSA039 and ΦMR003. The titers of phages are indicated as fold changes against the control and presented as means ± SD (n = 3). Significance against the control was analyzed by Tukey’s test based on one-way ANOVA: *p < 0.05, **p < 0.01. (**I**) Neutralization activity of purified anti-phage IgG against *Pseudomonas* phages ΦS12-3 and ΦR18 and *Escherichia* phages T1, T4, and T7. The titers of phages are indicated as fold changes against the control and presented as means ± SD (n = 3). Significance against the control was analyzed by *t* test. (**J–L**) Immunoelectron micrographs of ΦSA012, ΦSA039, and ΦMR003 stained with a gold-conjugated secondary antibody. White bars represent 100 nm. The size of the gold particles is 12 nm and black spots in the images represent gold particles conjugated to the secondary antibody.

To assess the immune response against phages, ΦSA012 was inoculated through s.c. injections twice as shown in Fig. 5A. Serum samples collected 14 and 28 dpi showed that IgG was induced significantly in mice compared to day 0, but the IgE level did not increase significantly during immunization (Fig. 5B and C). Furthermore, ELISA assays using ΦSA012 coated as an antigen showed that anti-phage antibody was significantly induced at 28 dpi, whereas serum samples at 48 hpi obtained from ΦSA012 i.p.-injected mice resulted in no immunological reaction (Fig. 5D).

We next assessed the neutralization activity of anti-phage antibody against ΦSA012. Figure 5E clearly shows that 1 µg/mL purified anti-phage IgG neutralized ΦSA012 (10^7^ pfu/mL) dramatically and decreased its titer to less than 10^5^ pfu/mL. In addition, 0.5, 0.25, and 0.125 µg/mL purified anti-phage IgG attenuated the plaque-forming activity of ΦSA012 from 90 to 50% (Fig. 5E). Lytic curves of antibody-treated ΦSA012 revealed that 0.5, 0.25, and 0.125 µg/mL antibody delayed the decrease of OD values compared to the control, but they reached the same OD values as the control after 8 hpi (Fig. 5F); however, 1.0 µg/mL antibody-treated ΦSA012 never showed efficient lytic activity. Furthermore, we investigated antibody specificity against multiple phages (10^7^ pfu/mL). Among *Staphylococcus* phages, purified anti-phage IgG harbored cross-reactivity against ΦSA039, which is classified as a similar *Staphylococcus* K-like virus similar to ΦSA012 (Fig. 5G). In addition, ΦMR003, which is a *Staphylococcus* virus, but of a different species than ΦSA012, was not strongly attenuated by the antibody compared to ΦSA012 and ΦSA039 (Fig. 5H). Moreover, the anti-phage antibody induced by ΦSA012 immunization resulted in no neutralization activity against *Pseudomonas* viruses such as ΦS12-3, *Myoviridae,* ΦR18, *Podoviridae*, or against *Escherichia* viruses such as T1, *Siphoviridae*, T4, *Myoviridae*, T7, *Podoviridae* (Fig. 5I).

We further analyzed the target of the anti-phage antibody towards *Staphylococcus* viruses such as ΦSA012, ΦSA039, and ΦMR003. Immuno-electron microscopic analysis clearly showed that the anti-phage antibody derived from ΦSA012-immunized mice recognized the long-tail fiber structure of ΦSA012 (Fig. 5J). For ΦSA039 and ΦMR003, colloidal gold-label signals were also clearly detected in long-tail fiber structures (Fig. 5K and L). To verify the effects of a secondary antibody and colloidal gold-labeled antibody on adsorption to ΦSA012, ΦSA039, and ΦMR003, we checked the viral titers after incubation with these phages and antibodies. Fig. S2 clearly shows that isotype control of the secondary antibody and colloidal gold-labeled antibody had no effects on the plaque-forming activities of the three phages.

### Therapeutic effect of ΦSA012 in a mouse infection model

**Figure 6:**
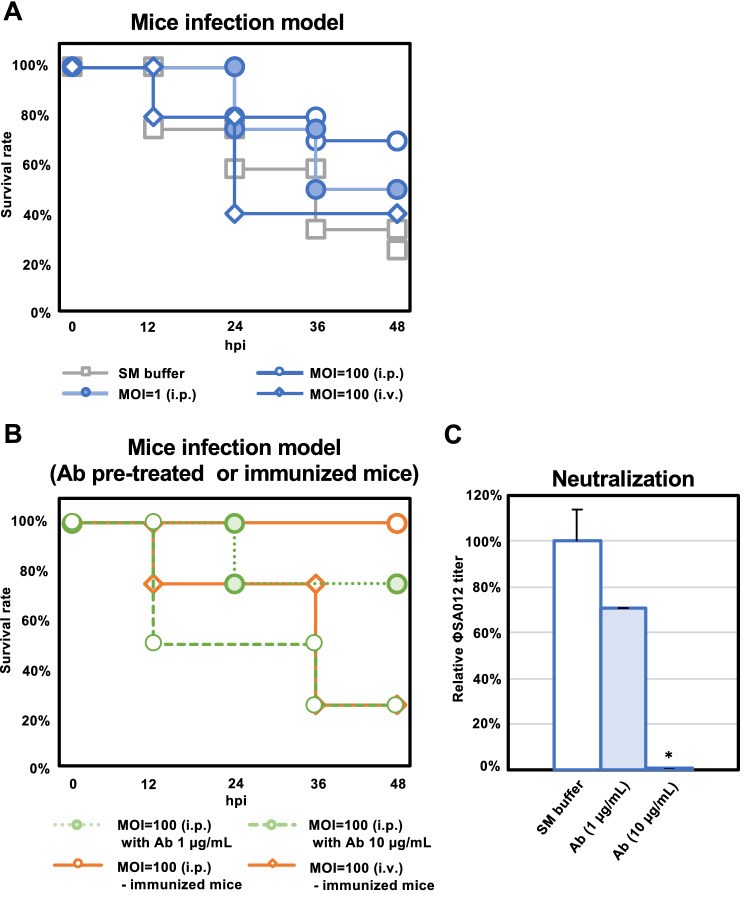
Therapeutic effect of ΦSA012 in a mouse infection model. (**A**) Survival rates of mice inoculated with SA003 through i.p. injections without ΦSA012 treatment (i.p. or i.v.). ΦSA012 was inoculated to mice i.p. or i.v. (n = 12 in the control group, n = 10 at MOI of 100 in the i.p. group, n = 10 at MOI of 100 in the i.v. group, n = 4 at MOI of 1 in the i.p. group). In every experiment, ΦSA012 or SM buffer was injected into mice 15 min after SA003 inoculation. (**B**) Survival rates of mice inoculated with SA003 through i.p. injection with anti-phage antibody (1 µg/mL or 10 µg/mL)-pretreated ΦSA012 treatment and ΦSA012 treatment in immunized mice (n = 4 in each group). ΦSA012 was injected into the mice 15 min after SA003 inoculation. (**C**) Neutralization activity of purified anti-phage IgG against ΦSA012 in vitro (equivalent to MOI of 100 in the mouse infection model). Titers of ΦSA012 are indicated as fold changes against the control and presented as means ± SD (n = 3). Significance against the control was analyzed by Tukey’s test based on one-way ANOVA: *p < 0.05, **p < 0.01.

In a mouse infection model using SA003, the survival rate without phage treatment was around 20% at 48 dpi (Fig. 6A). In contrast, i.p. injection of ΦSA012 to this model increased the survival rate up to 50% and 75% at 48 dpi at an MOI of 1 and 100, respectively. However, in the case of ΦSA012 i.v. injection at an MOI of 100, the survival rate was only 40%.

Finally, we investigated the effects of the antibody against ΦSA012 in the mouse infection model. First, we evaluated the effects of pretreatment with the anti-phage antibody against ΦSA012 on the survival rate in the mouse infection model. As shown in Fig. 6B, when 1 µg/mL anti-phage antibody-pretreated phages were i.p. injected into SA003-inoculated mice, the survival rate was around 75%, the same as that in ΦSA012-treated mice at an MOI of 100 by i.p. injection; however, i.p. injection of 10 µg/mL anti-phage antibody-pretreated ΦSA012 showed no therapeutic effect on SA003-inoculated mice. As shown in Fig. 6C, 1 µg/mL and 10 µg/mL anti-phage antibody treatments against ΦSA012 (5 × 10^9^ pfu/mL, the dose used for MOI of 100) revealed that plaque-forming activity in vitro was down to 70% and 0.01% compared to buffer control, respectively. As serum IgG levels of phage-immunized mice were around 600 µg/mL as shown in Fig. 5B, which was much higher than the pretreatment antibody concentration shown in Fig. 6C, we speculated that phages administered into the immunized mice would be neutralized by the anti-phage antibody in vivo. We thus evaluated the effects of ΦSA012 on therapeutic outcomes in phage-immunized mice. As shown in Fig. 6B, ΦSA012 (MOI of 100) i.p. injection after SA003 inoculation by i.p. resulted in a 100% survival rate in the phage-immunized mice, which might have been caused by insufficient contact between the anti-phage antibody and ΦSA012 (i.p.). However, when ΦSA012 (MOI of 100) was injected by i.v. into the phage-immunized mice, the survival rate decreased to 25%, which was less than that with ΦSA012 (MOI of 100) injection by i.v. into non-immunized mice.

## Discussion

Phage therapy is generally implemented by using cocktails composed of a variety of phages^11–13,24^. The type of phage cocktail is divided into two categories, personalized and fixed. Personalized phage cocktails have been applied in previous successful phage therapy cases^11–13^, and the Belgium medical system is now permitted to use personalized phage cocktails as magistral preparations^14^. This might be the best choice for treating bacterial infections individually as an order-made therapy; however, it is difficult to make personalized cocktails one by one as commercially marketed pharmaceuticals and in fact fixed cocktails are prepared in the Eliava Phage Therapy Center^24^. Thus, fixed phage cocktails are required instead of personalized cocktails and should cover a wider range of pathogenic strains. However, phages with narrow host ranges limit the efficacy of fixed cocktails or necessitate a larger number of constituent phages in the cocktail. Therefore, phages with wider host ranges are important for making versatile cocktails with a reduced number of phages required. In this context, the *Staphylococcus* virus K-like phage ΦSA012 (Fig. 1) has enormous potential for phage cocktail construction, as this phage possesses a wider host range against staphylococci as shown in Fig. 2. This result supports previous reports describing that *Staphylococcus* virus K-like phages exhibit a wider host range at the species level^15–17^. ΦSA012 recognizes WTA, which is widely conserved in staphylococci, as a receptor^19–21^ and has no *Sau*3A, which is a DNA restriction enzyme from *S. aureus*, recognition sites in its genome^25^. In addition, *S. aureus* has lost the clustered, regularly interspaced, short palindromic repeats (CRISPR)^25^, which might contribute to the wider host range of ΦSA012. Notably, ΦSA012 is active against almost all tested animal-associated *S. aureus*, suggesting that it is a promising and highly versatile phage for veterinary medicine; however, it is notable that ΦSA012 had a relatively narrow host range against human-associated MRSA (Fig. 2) compared to animal-associated staphylococci. Despite this, it has been previously reported that ΦMR003 possesses a wider host range against community-associated MRSA^26^, indicating that a phage cocktail composed of ΦSA012 and ΦMR003-like phages may cover several *S. aureus* pathogens. As shown in Fig. 3G, although other phage particles had no effect on ΦSA012 lytic activity, we need to further evaluate the effects of storage conditions on ΦSA012-containing phage cocktails, because a previous paper suggested that phage cocktail lytic activity might be attenuated by agglutination among constituent phages^27^, which is also why we should reduce the number of phages in any given cocktail.

Phages used in clinical applications need to be stable against various environmental factors. It has previously been reported that many phages have sufficient stability against heat and pH stress^28–30^. Similar to these reports, as shown in Fig. 3, ΦSA012 was resistant to several environmental conditions, suggesting that it has sufficient stability for its application in clinic. Notably, ΦSA012 showed stability against pH, with an optimum pH range of 3–9, suggesting that it would be inactive against target strains through oral administration given the acidic conditions in the gastrointestinal tract. Therefore, phage protection from severe acidic conditions, such as by using encapsulation, will be required to yield maximum therapeutic outcomes in oral administration. In addition, ΦSA012 injection into systemic circulation is a possible therapeutic route, as FBS treatment had no negative effect on ΦSA012 infectivity, and i.p/i.v injection did not result in any side effects such as inflammation or other pathologies (Fig. 3E, F and Fig. 4D). As shown in Fig. 3F, ΦSA012 produced higher number of plaques compared with control in inactivated-FBS treated groups, suggesting that enhanced growth activity of host cells by FBS might affect plaque forming activity of phages. In the case of i.p. injections into mice (Fig. 4A), phages spread through systemic circulation rapidly and reached the same levels as with i.v. injection of phages at 6 h, which is consistent with previous reports describing the immediate distribution of phages into systemic circulation and maximum phages counts in blood at 6 hpi in i.p. models^31–33^. However, ΦSA012 was diminished from serum within 48 h (Fig. 4A), suggesting that we need to administer phages to maintain their concertation in systemic circulation around once or twice per day, as previous studies suggest that successful phage therapy mainly depends on the dose^34,35^. In this context, it is useful to know that the order of magnitude of the present administrations, 10^9^ pfu/head (equivalent to MOI 100), did not cause any side effects. In addition, regarding storage conditions, ΦSA012 was stable for months at 4 °C (Fig. 3A), but investigation of additional storage conditions such as use of lyophilization or glycerol might be necessary based on previous reports^36,37^ if further long-term storage of ΦSA012 is required.

Phages never exhibit migratory activity toward target bacteria after administration in the body. Therefore, effective phage and target bacteria contact is important. Once phages contact the target bacteria, they can increase in number locally, which is a major advantage compared with classical antibiotics. However, our previous study demonstrated significant differences in the reduction rate of target bacteria depending on the route of administration^22^, indicating that it is necessary to consider efficient phage delivery based on their in vivo dynamics. In fact, i.v. injection of ΦSA012 decreased the survival rate compared with i.p. injection in the mouse infection model (Fig. 6A), suggesting that it took a long time to contact i.v.-injected phages with i.p.-injected SA003, leading to severe mortality. We also observed that ΦSA012 accumulated in the spleen and liver regardless of the route of administration (Fig. 4B and C). It has been reported that relatively high numbers of phages are detected in the spleen and liver with other routes of phage administration^38^, suggesting that phages can more efficiently contact target bacteria in these organs than in others. Staphylococci are well known to cause skin diseases such as atopic dermatitis in humans and pyoderma in dogs^39–41^. Skin diseases involve fewer physical barriers when compared with diseases in other tissues and thus might be better targets for phage therapy since they may allow more effective contact with target bacteria. As staphylococci shows critical virulence against the skin barrier and promotes atopic dermatitis^39,40^, this is an attractive target disease for phage therapy using ΦSA012. In the case of canine skin diseases, virulent staphylococci are not limited to *S. aureus*. It has been reported that *S. pseudointermedius* (SP) is often isolated from canine pyoderma^41,42^. As shown in Fig. 2, ΦSA012 has a wide host range but is not active against SP, and to our knowledge, virulent phages against SP have not yet been reported; however, it has been reported that ΦSA012-derived endolysin, which is a peptidoglycan hydrolytic enzyme, has a wider host range including SP, suggesting that the use of endolysin can complement ΦSA012-based phage therapy^43,44^.

Clinical cases of phage therapy have suggested that phage administration induces anti-phage antibodies^11,45,46^. In the present study, we showed that s.c. inoculation to mice with a high dose of ΦSA012 (10^11^ pfu/head) induced anti-phage antibodies (Fig. 5D). In our experiments, inoculation with phages alone was sufficient to elicit an immune response, which might be associated with the phage adjuvant activity^47^. In addition, the induced antibody reduced the plaque-forming activity of ΦSA012 in vitro (Fig. 5E and F), suggesting that they can neutralize the infectivity of the phage. The antibody induced by ΦSA012 also showed neutralizing activity against ΦSA039, which is closely related to ΦSA012 and is classified as a K-like virus^18,21,48^, and relatively weak neutralizing activity against ΦMR003, a different *Staphylococcus* virus species of the genus *Silviavirus*^26^ (Fig. 5G and H); however, the anti-phage antibody induced by ΦSA012 had no neutralizing activity against *E. coli* phages or *P. aeruginosa* phages (Fig. 5I), indicating that once an anti-phage antibody is induced by a specific phage, it might not affect the infectivity of other phages against distinct bacterial genera. Notably, electron microscopic analysis suggested that the anti-phage antibodies recognized and bound to the long-tail fibers of *S. aureus* phages (Fig. 5J–L), indicating that the infectivity of phages might be neutralized by inhibiting their adsorption. As the amino acid sequences and conformation of the long-tail fibers among *Staphylococcus* phages and other phages differ greatly, the neutralizing antibody against ΦSA012 could not neutralize *E. coli* phages or *P. aeruginosa* phages. Furthermore, as shown in Fig. 6A and B, the survival rate was the same as that of the phage-nontreated group when phages (equivalent to MOI 100) were pretreated with 10 µg/mL of the anti-phage antibody, since the titer of the phages was reduced to less than 0.1% at this time (Fig. 6C), suggesting that the effect of the anti-phage antibody on survival is small if there is no neutralization activity under conditions where the titer is reduced by more than 100 times, while administered phages would be neutralized in the bodies of immunized mice, because serum IgG levels of phage-immunized mice were around 600 µg/mL (Fig. 5B). Furthermore, as shown in Fig. 6B, ΦSA012 (MOI of 100) administered i.v. into phage-immunized mice did not rescue the survival rate, which was less than that by ΦSA012 (MOI of 100) i.v. injection into non-immunized mice (Fig. 6A). These results suggested that a high-dose ΦSA012 inoculation can induce a sufficient amount of anti-phage antibody to diminish the potent therapeutic effects in the present model; however, the effects of anti-phage antibodies is negligible in the case of actual human phage therapy because the induction of an anti-phage antibody depends on the route and dose of administration^45^. Notably, it has been suggested that the dose of phages in humans (ranging from 3 × 10^7^ to 6 × 10^10^ pfu/patient/day orally or topically), which is significantly lower than that used in the animal immunization model, results in too low induction of anti-phage antibodies^45,49^. As shown in Fig. 5D, anti-phage antibodies were not induced via i.p. injection significantly at 48 hpi as generally IgG is produced after around 14 days of immunization as described Fig. 5B, suggesting that phages show efficient lytic activity without neutralization in these periods. On the other hand, the level of anti-phage antibodies at 14 dpi and 28 dpi should be addressed after consecutive treatment of phages (i.e. twice a week via i.v. or i.p. injection) to obtain more detailed information for clinical application of phages in further analysis. It will be also necessary to be careful in the future to ensure that these antibodies do not induce autoimmune diseases when antibodies can be produced against phages. Nonetheless, we need to carefully address the dosage of phage administration given the possibility that anti-phage antibodies may be induced when ΦSA012 is applied in clinical situations. In addition, it would be of great utility to verify how much a patient’s serum harbors phage-neutralizing activity when phages are administered for long periods.

Previous studies well documented that phages are one of the promising approaches against AMR infections. Present study supports this approach as well; however, in fact, bacterial phage resistance has emerged during actual phage therapy cases^11,24,50^. Phage therapy thus faces problems similar to antibiotics. In order to address this issue, new strategies, which reverse antibiotic resistance, have been focused^50^. Actually, we have been previously reported that a type of *P. aeruginosa* variant acquires phage resistance and loses multi-drug efflux system MexXY-encoding gene at the same time, leading to restoration of fluoroquinolone sensitivity instead of a loss of phage sensitivity^51^. Similar with this strategy, restoration of antibiotics sensitivity of MRSA by ΦSA012 is expected through further analysis. Moreover, it has been also reported that antibiotics resistance bacteria change antibiotics sensitivity in antibiotic-free conditions^52^, which is one of the simple ways to make phenotypic conversion. Therefore, ΦSA012 efficiency against these kinds of antibiotics sensitivity reversed and non-reversed strains may provide new insight into future phage therapy.

In this study, we assayed the biological properties of the *Staphylococcus* virus ΦSA012 and found that it possesses promising characteristics for therapeutic clinical use, though we need to carefully study how to administer phages with the optimal dose and periods. These results provide insights into the use of *Staphylococcus* viruses for phage therapy to combat staphylococcal infections.

## Methods

### Staphylococcal strains and bacteriophages

*Staphylococcus aureus* clinical isolate SA003, which was isolated from a bovine mastitis case^18,22^, was used in the present study. *Staphylococcus* virus ΦSA012 used in the present study was previously isolated from sewage water collected from a sewage treatment plant (Tokyo, Japan) using plaque assays with SA003 as the host strain. The whole genome of ΦSA012 was also sequenced previously and submitted to DDBJ/EMBL/GenBank databases under accession number NC_023573.1. A phylogenetic tree of the phage whole genome was constructed by VICTOR^53^ using this phage sequence. In addition, *Staphylococcus* viruses ΦSA039^18,21,48^ and ΦMR003^26^ and *Pseudomonas* viruses ΦS12-3^51,54^ and ΦR18^54,55^, which were isolated and characterized previously, were used in this study. *Escherichia* viruses T1-T7 were obtained from the National Institute of Technology and Evaluation Biological Resource Center (NBRC, Chiba, Japan).

To reevaluate the host range of ΦSA012, 29 *S. aureus* veterinary isolates were obtained from mastitis cows^18,22^, 6 MRSA veterinary isolates were obtained from veterinary staff^56,57^, 40 MRSA human isolates were from the Kyoto Prefectural University of Medicine^58^, and 24 staphylococcal strains other than *S. aureus* were purchased from the Japan Collection of Microorganisms (JCM, Tsukuba, Japan) and used in the efficacy of plating (EoP) assay as described below. These strains are summarized in Table S1. For the isolation and use of human-derived MRSA clinical isolates, the experiments were approved by the Ethics Committee of Kyoto Prefectural University of Medicine, permit number ERB-C-1174-2.

### Bacteriophage preparation and plaque assay

For downstream assays, phages were propagated by the plate lysate method as described elsewhere^18^. In brief, an aliquot of the host or propagating strains (SA003, MRSA2007-13, *Pseudomonas aeruginosa* Pa12, and *Escherichia coli* B4T strain purchased from NBRC) grown in Luria–Bertani (LB) medium (Becton Dickinson, Franklin Lakes, NJ, USA) was combined with an aliquot of phage (ΦSA012, ΦSA039, ΦMR003, ΦS12-3, ΦR18, and T1-T7 phages) and added to 3 mL of LB top agar containing 0.5% agarose ME (Iwai Chemicals Company, Tokyo, Japan). The mixture then was overlaid on an LB agar plate. After overnight incubation of the plate at 37 °C, 3 mL of SM buffer (10 mM MgSO_4_, 100 mM NaCl, 0.01% gelatin, and 50 mM Tris–HCl [pH 7.5]) was added to the plate, and the plate was incubated at room temperature for 1–2 h with shaking. The overlaid top agar was scraped off and homogenized with SM buffer using a colony spreader. The collected homogenate was centrifuged at 6,500 g for 15 min at 4 °C to remove remaining bacteria and debris. The resultant supernatants were passed through 0.45-µm membrane filters (ADVANTC, Tokyo, Japan) and purified using Amicon Ultra-membrane filters (Merck, Darmstadt, Germany) based on the phage on tap (PoT) method as described by Bonilla et al.^59^*.*

High-titer ΦSA012 stock for mice immunization was propagated by infecting 400 mL of exponentially growing SA003 at multiplicity of infection (MOI) of 0.1 in LB medium. After 8 h incubation with shaking, 1 M NaCl was added and incubated on ice for 1 h. Thereafter, culture medium was centrifuged at 10,000 g for 30 min at 4 °C. The resultant supernatant was vacuum-filtered by a 0.45-µm membrane filter then mixed with 10% (w/v) polyethylene glycol 6000 (PEG6000, Nacalai Tesuque, Kyoto, Japan) and incubated at 4 °C overnight. ΦSA012 was pelleted by centrifugation at 10,000 g for 90 min at 4 °C and resuspended in SM buffer and chloroform (Wako Pure Chemical, Tokyo, Japan). Thereafter the aliquot was centrifuged at 8,000 g for 10 min at 4 °C. The resultant supernatant was harvested and further concentrated by the PoT method as described above. The phage titer was calculated as the number of plaques in a plaque assay using host or propagating strains, in accordance with previous reports, and is represented as plaque-forming units per milliliter (pfu/mL).

### Efficacy of plating (EoP) assay

Phages host range determination was performed by the EoP method in accordance with previous reports^23^. In brief, staphylococcal strains were grown in LB medium overnight at 37 °C with shaking. 3 mL of LB top agar containing 100 µL of staphylococcal strains was overlaid on an LB agar plate. Thereafter, 3 µL of diluted ΦSA012 aliquot (10^7^ to 10^1^ pfu/mL) in SM buffer was dropped onto an overlaid LB agar plate to observe the lytic activity of ΦSA012 by plaque formation. EoP values represent pfu using the specific staphylococcal strain/pfu using SA003.

### Monitoring of phage lytic activity by a plate reader

The lytic activity of ΦSA012 against SA003 under several conditions was evaluated in turbidity assays by monitoring the OD_590_ for 24 h using a plate reader (Sunrise Rainbow Thermos RC, TECAN, Austria) as previously reported^51^. In brief, ΦSA012 was inoculated into exponentially growing SA003 cultures in a 96-well plate at a multiplicity of infection of 0.01. The density of the culture was monitored using a plate reader every 1 h.

### Phage stability against multiple environmental conditions

The effects of storage conditions on plaque-forming activity were evaluated after incubation of ΦSA012 at room temperature for 2, 7, and 31 days or at 4 °C for 30 and 60 days. In addition, the freeze–thaw effect on ΦSA012 was evaluated by incubation at − 80 °C for 24 h and 37 °C for 5 min for five cycles. To assess phage stability against temperature, ΦSA012 was incubated at 37 °C, 56 °C, and 60 °C for 1, 5, 24, 72, and 168 h. To evaluate ΦSA012 resistance to pH, phages were exposed to a gradient pH buffer ranging from 1 to 11 for 1 h. To assess the effects of serum against phages, ΦSA012 was mixed with fetal bovine serum (FBS, Biowest, Nuaille, France) at concentrations of 5%, 10%, and 20% with or without inactivation at 56 °C for 30 min. ΦSA012 (10^7^ pfu/mL) in SM buffer was used in these experiments. To evaluate the effects of other phage particles against plaque-forming activity, ΦSA012 in SM buffer (10^5^ pfu/mL) was mixed with corresponding selected T1-T7 phages (10^3^ or 10^5^ pfu/mL). After these treatments, the ΦSA012 titer was evaluated by plaque assay as described above. In addition, phage lytic activities after several environmental treatments were also detected by a plate reader as described above.

### Animals

This study was carried out in strict accordance with the recommendations in the Guide for the Care and Use of Laboratory Animals of the National Institutes of Health and ARRIVE guidelines. All experiments were performed in accordance with relevant guidelines and regulations. The protocol was approved by the Ethics Committee for Animal Experiments of Rakuno Gakuen University (Permit Number: VA20A10). Healthy and specific pathogen-free 5–8-week-old ddY mice purchased from Japan SLC (Shizuoka, Japan) were fed and housed in filter-top cages in an air conditioned animal facility under a 12 h/12 h light–dark cycle and allowed to adapt to their environment for one week.

### Pharmacokinetics of ΦSA012

The mice were injected with ΦSA012 (5 × 10^9^ pfu/head) intraperitoneally (i.p.) or intravenously (i.v.). After 0.25, 3, 6, 12, 24, and 36 h post-inoculation (hpi), blood samples were collected from the caudal veins of mice. At 48 hpi, organ and blood samples were collected from mice under medetomidine/midazolam/butorphanol anesthesia. After dissection, the excised samples were weighed and divided into two samples: one was fixed with 4% paraformaldehyde for 14 h for hematoxylin and eosin (HE) staining, and the other was homogenized in phosphate buffered saline (PBS). After homogenization, the samples were centrifuged at 900 g for 10 min at 4 °C. Obtained blood samples were also centrifuged at 900 g for 25 min at 4 °C. The resultant supernatants were used for plaque assays using SA003 as described above.

### Hematoxylin and eosin (HE) staining

Fixed organ samples were dehydrated with 70–100% ethanol, cleaned in xylene, and embedded in paraffin wax. Serial Sections (4 µm) were stained with hematoxylin and eosin and observed using an FSX100 microscope (Olympus, Tokyo, Japan).

### Mouse immunization model

Mouse immunization was performed in accordance with a previous report with some modifications^60^. In brief, mice were injected with ΦSA012 (10^11^ pfu/head) subcutaneously (s.c.). Two weeks after the first inoculation (14 days post-inoculation [dpi]), the mice were injected with ΦSA012 (10^11^ pfu/head) s.c. again. Blood samples collected at 14 dpi and 28 dpi were centrifuged at 900 g for 25 min at 4 °C, and then sera were stored at − 30 °C until use. IgG was purified from serum samples collected at 28 dpi using a spin column-based Antibody Purification Kit–Protein G (Cosmo Bio, Tokyo, Japan) according to the manufacturer’s protocols. Purified IgG concentrations were determined by enzyme-linked immunosorbent assay (ELISA) as described below.

The neutralization activity of purified IgG was measured against ΦSA012, ΦSA039, ΦMR003, ΦS12-3, ΦR18, T1, T4, and T7 in vitro. In brief, phages (10^8^ pfu/mL or 5 × 10^10^ pfu/mL) were mixed with purified IgG and SM buffer and incubated for 2 h at room temperature. Thereafter, aliquots were used for plaque assays or monitoring lytic activity using a plate reader as described above.

### Enzyme-linked immunosorbent assay (ELISA)

IgG, IgM, and IgE levels in the sera of immunized mice were determined using an Uncoated IgG, IgM, and IgE ELISA kit (Invitrogen, Carlsbad, CA, USA) in accordance with the manufacturer’s protocols. Absorbance was measured at 490 nm by an iMARK microplate reader (BioRad, Hercules, CA, USA).

To determine the immunoreactivity of anti-phage antibody in serum from ΦSA012-immunized mice, we performed ELISA for ΦSA012 based on a previous report^60^. ΦSA012 purified by the PoT method was further purified by CsCl ultracentrifugation at 40,000 g for 1 h at 4 °C as described elsewhere. Nunc-immuno plates (Nunc, Roskilde, Denmark) were coated with CsCl ultracentrifugated-ΦSA012 (1.0E + 10 pfu/well) overnight at 4 °C. Thereafter, the plates were blocked with 0.5% gelatin in PBS for 2 h at room temperature. Serum samples from immunized mice were added to the plates and incubated for a further 2 h at room temperature. After washing with 0.05% Tween 20 PBS three times, a secondary antibody against mouse IgG conjugated with alkaline phosphatase (Merck, Darmstadt, Germany) was added to the plate at a dilution of 1:5000 and incubated for 1 h at room temperature. After washing with 0.05% Tween 20 PBS three times, the plate was incubated with 100 µL of *p*-nitro-phenyl phosphate (Tokyo Chemical Industry, Tokyo, Japan) for 30 min at room temperature, and then absorbance was measured at 405 nm by an iMARK microplate reader.

### Immunoelectron microscopic analysis

Immunoelectron microscopic analysis was performed as described previously^19^. Purified ΦSA012, ΦSA039, and ΦMR003 aliquots by CsCl ultracentrifugation (10^10^ pfu/mL) were mixed with purified IgG from ΦSA012-immunized mice (1.0 µg/mL) and incubated for 2 h at room temperature. The samples were loaded onto ester-carbon-coated copper grids (EM Japan, Tokyo, Japan). After 10 min incubation at room temperature, the copper grids were washed with SM buffer three times and then incubated with 12-nm colloidal gold-labeled donkey anti-mouse IgG (H + L) (Jackson Immuno Research Laboratory, West Grove, PA, USA) for 30 min at room temperature. After washing with SM buffer three times, the grids were stained with 2% uranyl acetate. Stained samples were observed with a Hitachi HT7700 transmission electron microscope (Hitachi, Tokyo, Japan) at 75 kV.

### Mouse infection model

The mice were inoculated with SA003 at 5 × 10^7^ cfu/head through i.p. injection. After a 15-min interval, ΦSA012 (5 × 10^9^ pfu/head for MOI of 100 or 5 × 10^7^ pfu/head for MOI of 1) was inoculated through i.p. or i.v. injection. An equal amount of SM buffer was administered to the control group.

### Statistical analysis

Statistical analysis was performed using *t* tests to compare two groups and Tukey’s test based on one-way ANOVA to compare three or more groups from at least three experiments. *p* values of less than 0.05 were considered statistically significant. Where applicable, statistical significance is indicated by asterisks, with *p < 0.05 and **p < 0.01. All statistical analyses were performed using Microsoft Excel ver. 16.63.

## Supplementary Information

Supplementary Information 1.

Supplementary Information 2.

## Data Availability

The datasets generated during and/or analysed during the current study are available from the corresponding author on reasonable request and DNA sequences of the phage are available DDBJ/EMBL/GenBank databases under accession number NC_023573.1.
