# Supplementary material for: Biological properties of Staphylococcus virus ΦSA012 for phage therapy
Source: Sci Rep. 2022 Dec 9;12:21297. doi: 10.1038/s41598-022-25352-6 (PMC9734660; doi:10.1038/s41598-022-25352-6)
Supplement: Supplementary file 1 — Supplementary Information 1. [file 41598_2022_25352_MOESM1_ESM.pdf]

Figure.S1

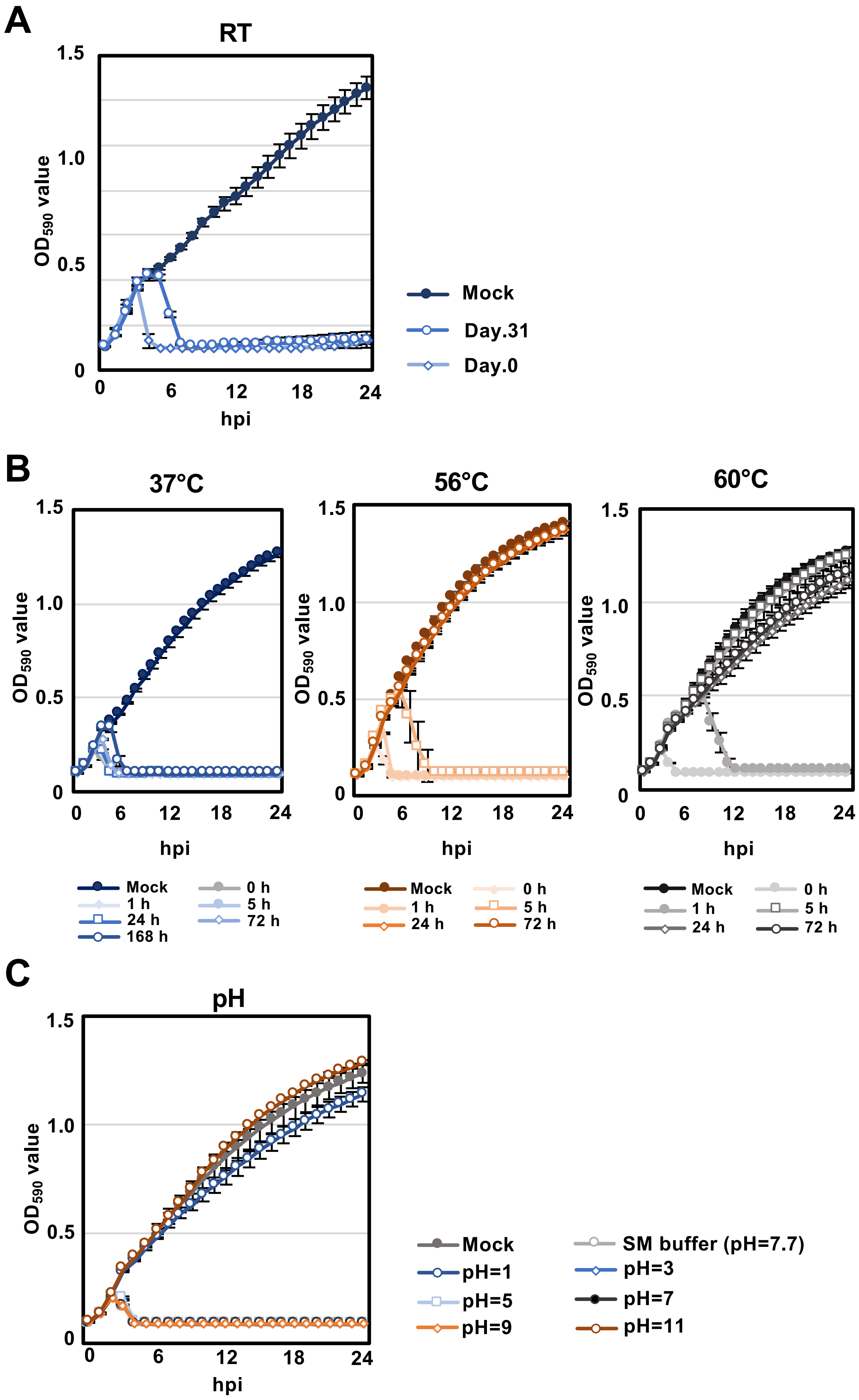
A
RT
1.5
1.0
0.5
0
OD590 value
0
6
12
18
24
hpi
Mock
Day.31
Day.0
B
37°C
56°C
60°C
hpi
Mock
1 h
24 h
168 h
0 h
5 h
72 h
C
pH
Mock
pH=1
pH=5
pH=9
SM buffer (pH=7.7)
pH=3
pH=7
pH=11

Figure.S1

Figure S1. Monitoring of ΦSA012 OD value after various environmental stimulations.
(A) Lytic curves of SA003 growing in the presence of ΦSA012 stored at room temperature and 4° C were obtained by monitoring the OD_{590} until 24 hpi. The individual points in each lytic curve are presented as means ± SD (n = 4). (B) Lytic curves of SA003 growing in the presence of ΦSA012 exposed to 37° C, 56° C, and 60° C for specific times were obtained by monitoring the OD_{590} until 24 hpi. The individual points in each lytic curve are presented as means ± SD (n = 4). (C) Lytic curves of SA003 growing in the presence of ΦSA012 exposed to different pH ranges were obtained by monitoring the OD_{590} until 24 hpi. The individual points in each lytic curve are presented as means ± SD (n = 4).

Figure.S2

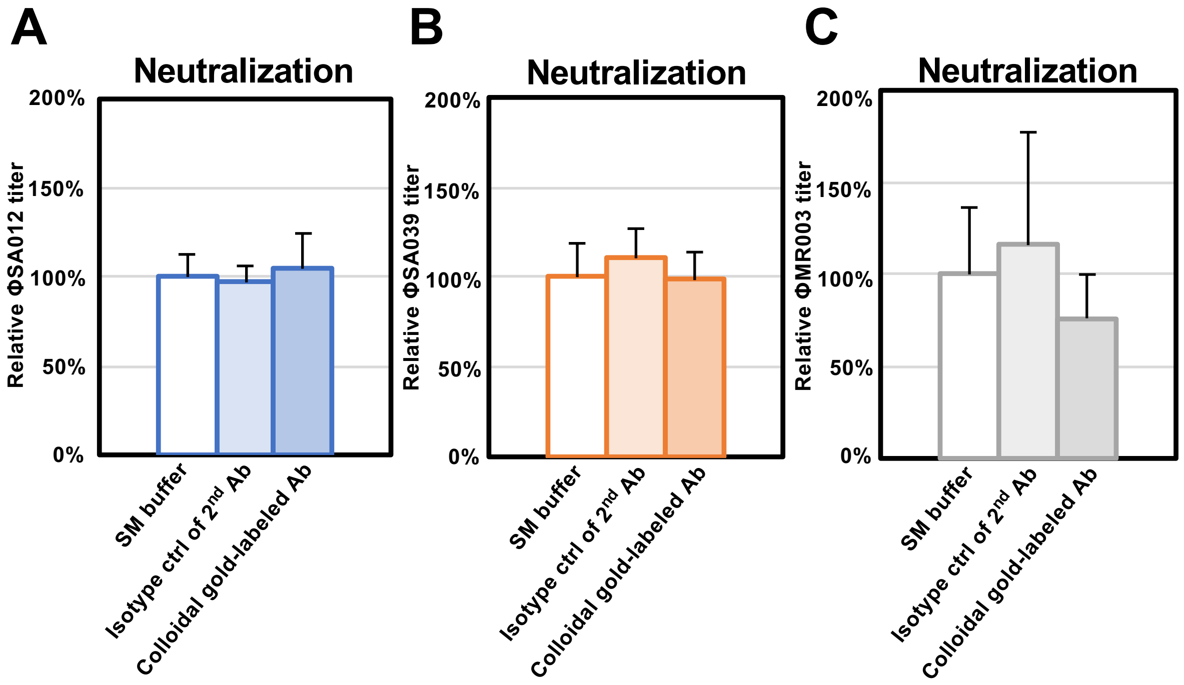

Figure S2. Effects of secondary and colloidal gold-labeled antibodies on phage plaque-forming activity.

(A) Effects of antibodies on the plaque-forming activity of ΦSA012. (B) Effects of antibodies on the plaque-forming activity of ΦSA039. (C) Effects of antibodies on the plaque-forming activity of ΦMR003. Titers of each phage are indicated as fold changes against the control (SM buffer treated) and presented as means ± SD (n = 3). Significance against the control at each condition was analyzed by Tukey's test based on one-way ANOVA.
